# Analysis of nitrate reductase mRNA expression and nitrate reductase activity in response to nitrogen supply

**Published:** 2014-06

**Authors:** Gholamreza Kavoosi, Sadegh Balotf, Homeira Eshghi, Hasan Hasani

**Affiliations:** 1 Institute of Biotechnology, Shiraz University, Shiraz, Iran; 2 Institute of Biotechnology, Guilan University, Guilan, Iran

**Keywords:** *Triticum aestivum*, nitrate, Potassium nitrate, Nitrate reductase

## Abstract

Nitrate is one of the major sources of nitrogen for the growth of plants. It is taken up by plant roots and transported to the leaves where it is reduced to nitrite in the. The main objective of this research was to investigate stimulatory effects of sodium nitrate, potassium nitrate, ammonia and urea on the production/generation of the nitrate reductase mRNA in *Triticum aestivum *plants. The plants were grown in standard nutrient solution for 21 days and then starved in a media without nitrate for seven days. Starved plants were stimulated with various concentrations of sodium nitrate, potassium nitrate, ammonia and urea, and the expression of nitrate reductase mRNA was analyzed by real-time PCR. Our results indicated that starvation caused significant decrease in the production of nitrate reductase mRNA in the plant leaf. Sodium and potassium nitrate were capable of restoring the production of nitrate mRNA in a dose-dependent manner, since 50 mM of each produced the highest level of the mRNA. The stimulatory effect of potassium nitrate was higher than sodium nitrate, while ammonia and urea did not show such activity. At low concentrations, sodium nitrate and potassium nitrate caused significant increase in the nitrate/nitrite mRNA production, whereas high concentrations of these salts suppressed the expression of this gene considerably.

## INTRODUCTION

One of the major sources of nitrogen for plant growth is nitrate. Nitrate in the soil is taken up by plant roots, reduced to nitrite, stored in the vacuoles of root cells or transported to the leaves where it is reduced or stored in the vacuoles of leaves [1-3]. Nitrate reductase and nitrite reductase catalyze the reduction of nitrate to nitrite and nitrite to ammonia. Ammonia can enter amino acid and proteins via carbon skeletons produced by photosynthesis. Because of the available carbon skeletons provided by photosynthesis, the reduction of nitrate occurs more efficiently in leaves than roots [4-7]. In the leaf cytosol, nitrate is reduced to nitrite by nitrate reductase. Nitrite is transported to the chloroplasts and reduced to ammonia by nitrite reductase [8-15]. To the authors’ knowledge, the molecular mechanism of the nitrate reduction pathway in response to sodium nitrate, potassium nitrate, ammonia and urea is not clearly known yet.

To gain more insight on the nitrate reduction pathway we examined the effects of these salts on nitrate reductase mRNA expression and nitrate reductase activity in the leaves of wheat.

## MATERIALS AND METHODS

**Plant treatments: **The plants were grown in a standard nutrient solution (Hoagland solution) for 21 days. After that they were divided into two groups; one grown in a standard nutrient solution (control) and another grown in a media without nitrate for seven days (starvation). Starved plants were divided into five groups; (1) grown in media without nitrate for four days, (2) grown in various concentrations of potassium nitrate (25, 50 and 75 mM), (3) grown in various concentrations of sodium nitrate (25,50 and 75 mM), (4) grown in various concentrations of ammonia (25, 50 and 75 mM) and (5) grown in various concentrations of urea (25, 50 and 75 mM), all for three days. After 24 h, the leaves were harvested and frozen in liquid nitrogen until use.

**Nitrate reductase activity assay: **Frozen leaves were powdered with a mortar and pestle and solubilized in phosphate buffered saline pH 7.4. The extract was centrifuged at 6000 *g *for 15 min and the supernatant was stored at -70°C. Nitrate reductase activity was examined by the reduction of nitrate to nitrite [16, 17]. One unit of nitrate reductase activity is defined as the production of 1µ M nitrite per min. Specific activity is described as a unit of nitrate reductase activity divided by gram of total protein. Results were reported as percentages of control plants.

**RNA extraction and cDNA synthesis: **Total RNA was extracted using an RNA- plus (RNX-plus) buffer from Cinagen (Tehran, Iran). 500 mg powdered leaves were transferred to 1 mL RNA-plus buffer in RNase-free microtubes, mixed and left at room temperature for 5 min. 200 µ L of chloroform was added to the sample and mixed slowly. The mixture was centrifuged at 13200 *g *at 4°C for 15 min. The resulting supernatant was transferred to a new tube and precipitated with an equal volume of isopropanol for 15 min at 4°C. The RNA pellet was washed using 75% ethanol and solubilized in 15 µ l of RNase-free water. The quantification of purified total RNA was performed using a Nano-Drop ND 1000 spectrophotometer (Wilmington, USA). DNase treatment was carried out using a Fermentas DNase Kit (Fermentase, Hanover, MD) according to the manufacturer’s instructions. Five µ g of DNase-treated RNA was used for first strand cDNA synthesis, using a cDNA synthesis Fermentas Kit (Fermentase, Hanover, MD) according to the manufacturer’s instructions.

**Quantitative real-time PCR: **Primers in the form of exon Junction were designed using AlleleID 7 software (premier Biosoft Intl, Palo Alto, CA) for internal controls 18S rRNA (AY049040) and nitrate reductase (X57844) genes (Table 1). The primers’ sequence for were; 5′-CGC TCC TAC CGA TTG AAT GG-3′ (sense) and 5′-TCC TTG TTA CGA CTT CTG CTT CC-3′ (anti-sense). The primers’ sequence for nitrate reductase were; 5'-GGC AAC TTC GTC ATC AAC-3' (sense) and 5'-CAT CTC CGT CTC GTC CTC-3' (anti-sense). Relative real-time PCR was performed at 20 µ L volume containing 4 pM of each primer, 1 µ L cDNA and 1x Syber green buffer (Qiagen, Hilden, Germany). Amplification reactions were carried out in a line Gene K Thermal cycler (Bioer Technology Co, Hangzhou, China) with an initial denaturing of 94°C for 2 min, followed by 40 cycles of 94°C for 10s. The annealing temperature (Ta) of each primer was 58°C for 15s. The specificity of the amplifications was checked based on the melting curves resulting from heating the amplicons from 50-95°C. All amplification reactions were repeated twice under identical conditions. To ensure that PCR was generated from cDNA rather than genomic DNA, a proper control reaction was carried out without reverse transcriptase treatment. For quantitative real-time PCR, the relative expression of reductase genes was calculated based on the threshold cycle (CT) method. The CT for each sample was calculated using the Line-Gene K software [18]. The expression of target mRNA over the reference value was calculated by equation 2–ΔΔCT. ΔCT was determined by subtracting the internal control CT value from the specific CT of the test gene and ΔΔCT was obtained by subtracting the ΔCT of each experimental sample from the control sample [19].

**Statistical analysis: **Data were analyzed as a completely randomized design with three replications and expressed as means ± standard deviations. Significant differences between treatments were analyzed by one-way analysis of variances (ANOVA) and Duncan tests at P<0.05 using the statistical package for the social sciences (SPSS, Abaus Concepts, Berkeley, CA) software.

## RESULTS

**Nitrate reductase activity: **The activity of nitrate reductase in leaves of the control plants was 100%. Starvation caused a significant decrease in nitrate reductase activity to

50±2.4% of the control (Table 1). In the plants supplemented with 25, 50, and 75 mM sodium nitrate, enzyme activities were of the control, respectively (Table 1). In plants supplemented with 25, 50, and 75 mM potassium nitrate, enzyme activities were of the control, respectively (Table 1). In plants supplemented with 25, 50, and 75 mM ammonia, enzyme activities were of the control, respectively (Table 1). In plants supplemented with 25, 50, and 75 mM urea, enzyme activities were of the control, respectively (Table 1). Sodium and potassium nitrate restored nitrate activity while ammonia and urea did not show such activity.

**Table 1:** Effects of sodium nitrate, potassium nitrate, ammonia and urea on the nitrate reductase activity in the starved plants.

	** NaNO** **3**	**KNO** **3**	**NH4** **+**	**Urea**
Control	100 ± 4.5	100 ± 4.5	100 ± 4.5	100 ± 4.5
Starvation	50 ± 2.4	50 ± 2.4	50 ± 2.4	50 ± 2.4
25 mM	57 ± 4.8	102 ± 8.4	46 ± 1	43 ± 1
50 mM	150 ± 6.8	156 ± 8.5	43 ± 1.5	41 ± 1.2
75 mM	106 ± 6	144 ± 9	39 ± 1.4	38 ± 1.2

Plants were grown in standard nutrient solution for 21 days. Plants starved for 1 week, then used for

induction experiment with sodium nitrate, potassium nitrate, ammonia and urea (25, 50, 75 mM) for three days. Data are means ± standard deviation for three tests.

**Nitrate reductase specific activity: **The specific activity of nitrate reductase in leaves of the control plants was 100%. Starvation caused a significant decrease in nitrate reductase specific activity to of the control (Table 2). In the plants supplemented with

25, 50, and 75 mM sodium nitrate, enzyme specific activities were of the control, respectively (Table 2). In plants supplemented with 25, 50, and 75 mM potassium nitrate, enzyme specific activities were of the control, respectively (Table 2). In plants supplemented with 25, 50, and 75 mM ammonia, enzyme specific activities were of the control, respectively (Table 2). In plants supplemented with 25, 50, and 75 mM urea, enzyme specific activities were of the control, respectively (Table 2). Sodium and potassium nitrate restored nitrate reductase specific activity while ammonia and urea did not show such activity.

**Table 2:** Effects of sodium nitrate, potassium nitrate, ammonia and urea on the specific activity of nitrate reductase in the starved plants.

	**NaNO** **3**	**KNO** **3**	**NH4+**	**Urea**
Control	100 ± 4.8	100 ± 4.8	100 ± 4.8	100 ± 4.8
Starvation	40 ± 2.7	40 ± 2.7	40 ± 2.7	40 ± 2.7
25 mM	81 ± 4.4	89 ± 8.8	39 ± 2	35 ± 1.3
50 mM	129 ± 6	153 ± 7	42 ± 2.7	35 ± 2
75 mM	99 ± 4.5	102 ± 6	37 ± 1.3	34 ± 1.3

Plants were grown in standard nutrient solution for 21 days. Plants starved for 1 week, then used for induction experiment with sodium nitrate, potassium nitrate, ammonia and urea (25, 50, 75 mM) for three days. Data are means ± standard deviation for three tests.

**Nitrate reductase mRNA expression: **Nitrate reductase mRNA expression in the control plants was 100% while starvation caused a significant decrease in the mRNA expression to of the control (Table 3). In the plants supplemented with 25, 50, and 75 mM sodium nitrate, the expressions of nitrate reductase mRNA of the control, respectively (Table 3). In plants supplemented with 25, 50, and 75 mM potassium nitrate, expressions of nitrate reductase mRNA were of the control, respectively (Table 3). In plants supplemented with 25, 50, and 75 mM ammonia, expressions of nitrate reductase mRNA were of the control, respectively (Table 3). In plants supplemented with 25, 50, and 75 mM urea, expressions of nitrate reductase mRNA were of the control, respectively (Table 3). Thus, only sodium and potassium nitrate stimulated nitrate reductase mRNA production in the starved plants.

**Table 3:** Effects of sodium nitrate, potassium nitrate, ammonia and urea on the expression of nitrate reductase mRNA in the starved plants.

	**NaNO** **3**	**KNO** **3**	**NH4** **+**	**Urea**
Control	100 ± 4.4	100 ± 4.4	100 ± 4.4	100 ± 4.4
Starvation	43 ± 2.2	43 ± 2.2	43 ± 2.2	43 ± 2.2
25 mM	47 ± 4.3	79 ± 6.2	39 ± 1	37 ± 1
50 mM	147 ± 6.5	183 ± 11	36 ± 1	35 ± 1.3
75 mM	124 ± 5.2	132 ± 9	33 ± 1.2	33 ± 1.2

Plants were grown in standard nutrient solution for 21 days. Plants starved for 1 week, then used for induction experiment with sodium nitrate, potassium nitrate, ammonia and urea (25, 50, 75 mM) for three days. Data are means ± standard deviation for three tests.

## DISCUSSION

The aim of this study was to investigate the effects of sodium nitrate, potassium nitrate, ammonia and urea supplements on nitrate reductase activity and nitrate reductase mRNA expression in the nitrogen-starved wheat seedling. Our results clearly indicated that sodium and potassium nitrate significantly restored nitrate reductase activity and nitrate reductase mRNA expression in wheat leaves, while such activity was not observed for ammonia and urea. Nitrate in the soil is absorbed through nitrate transporters in root cells. A large portion of nitrate is transported to the leaves where it is either stored in the vacuoles or reduced to nitrite. Thus, nitrate levels in the leaves increase with nitrate supplements in the soil. The efficiency of the net nitrate uptake is highly regulated by endogenous nitrate with a negative feedback strategy [12-15, 20,

21]. When external nitrate is higher than the plant’s demand, the decrease in nitrate accumulation might be due to the decrease in nitrate uptake. Excessive nitrate supplements in the soil will finally cause a decrease in nitrate uptake, which will result in soil nitrate pollution [12-15, 22, 23]. At the same time, ammonium and glutamine can regulate nitrate uptake by a negative feedback strategy. Exposure of plants to nitrate induces nitrate transporters and nitrate uptake, since metabolites resulting from nitrate reduction (ammonium and glutamine or other amino acids), down-regulate nitrate transporters and nitrate uptake [20, 21, 24-27].

Urea in the soil is hydrolyzed by bacterial urease into ammonium, converted to nitrate and uptaked. A large portion of urea is directly uptaked by plant roots and hydrolyzed to carbon dioxide and ammonium. Thus, fertilization with urea will result in the simultaneous exposure of plant roots to urea, ammonium and nitrate. Accordingly, urea can probably repress nitrate influx through ammonium production since plants supplemented with urea and ammonia showed signs of nitrogen starvation. Reduced levels of nitrate reductase expression in the plants supplemented with urea and ammonia could therefore be related to the suppressive effects of ammonia [28-31].

Nitrite reductase activity, which reduces nitrate to nitrite, is a rate-limiting enzyme for nitrate reduction in higher plants. The activity of this enzyme increases in low nitrate concentrations but decreases at higher cytoplasmic nitrate levels [1-3, 32, 33]. The present study suggests that the relationship between nitrate reductase activity and nitrate reductase mRNA depends on the external nitrate. Low concentrations of external nitrate have a positive effect on nitrate reductase activity, since higher concentrations of external nitrate diminish the activity of nitrate reeducates [34-36]. Furthermore, our results indicated that only sodium and potassium nitrate at low concentrations can restore nitrate reductase mRNA expression/production in the starved plants. At higher concentrations of sodium and potassium nitrate, the expression of nitrate reductase mRNA significantly decreased, which could be related to negative feedbacks of ammonium, glutamine or other amino acids [37-39].

The major finding of this research was the different effects of potassium nitrate, sodium nitrate, ammonia and urea on nitrate reductase expression and activity. On this pathway, potassium nitrate was more effective than sodium nitrate, while ammonia and urea were ineffective. Although both potassium and sodium nitrate contain nitrate, they are different in terms of potassium and sodium cations. Thus, the differential activity could be related to the potassium and sodium contents. Potassium is an essential macronutrient for higher plants and serves functions such as enzyme activation, anions neutralization, pH regulation, and phloem transport and cell turgidity. Accordingly, the availability of potassium in the plant cell strongly regulates the rate of biochemical reactions [40, 41]. Potassium is important in regulating the rate of photosynthesis and ATP production by maintaining the membrane potential. In potassium deficient conditions, the rate of photosynthesis and ATP production reduces considerably, and all processes dependent on ATP are slowed down [42, 43]. Potassium is required for protein synthesis and the reading of genetic codes in the plant cells. It is also required for the elongation of peptide chains. Thus, when plants are deficient in potassium, proteins are not fully synthesized and truncated proteins accumulate in the cells [44,45]. In addition, sugars produced during photosynthesis must be transported to other parts of the plant for utilization and storage, and the availability of potassium and ATP production can help these transportation systems function normally [46-49]. Starch synthesis is also activated by potassium; in potassium deficient conditions, the level of starch declines since soluble carbohydrates accumulates. Under adequate potassium levels, soluble carbohydrates are efficiently moved from production sites and converted to starch [50-53]. Sodium, on the other hand, is not an essential element and cannot be expected to play any specific role in the metabolic activities of plants. In high concentrations, sodium might entirely replace potassium, but it is useless as a substitute for potassium since it reduces the positive physiological roles of potassium [54-57].

supplements stimulate nitrate reductase expression/activity at low concentrations of both external potassium and sodium nitrate; the potassium nitrate being more effective. More positive effects of potassium nitrate are probably due to the role of potassium in enzyme activity and expression. At high concentrations of external nitrate, nitrate reductase expression/activity reduced, which might be related to the suppressive effect of ammonium and other nitrate metabolites. Ammonia and urea supplementation did not restore the nitrate reductase expression/activity in the starved plants, which might be related to the negative feedback of ammonia towards the nitrate influx pathway.
